# Occupational health care personnel tackling alcohol overuse – an observational study of work processes and patient characteristics

**DOI:** 10.1186/s12889-021-12473-2

**Published:** 2022-01-11

**Authors:** Jarmo O. Kuronen, Klas Winell, Jelena Hartsenko, Kimmo P. Räsänen

**Affiliations:** 1 Etelä-Savon Työterveys Oy, Maaherrankatu 13, 50100 Mikkeli, Finland; 2 Conmedic Oy, Antaksenkuja 3, 02330 Espoo, Finland; 3 Department of Business Administration, Tallinn University of Technology, Ehitajate tee 5, 12616 Tallinn, Estonia; 4 University of Eastern Finland, Faculty of Health Sciences, School of Medicine, Institute of Public Health and Clinical Nutrition, Box 1627, 70211 Kuopio, Finland

**Keywords:** Alcohol, Alcohol use disorder, Brief intervention, Depression, Disability, Health check-up, Intervention, Occupational health care, Pension, Prevention

## Abstract

**Background:**

Overuse of alcohol is a significant risk factor for early retirement. This observational study investigated patient characteristics and work processes in occupational health care (OHC) affecting practices in tackling alcohol overuse.

**Methods:**

The data were from 3089 patient contacts gathered for quality improvement purposes in fifteen OHC units during the years 2013–2019 in Finland. A two-proportion z-test was performed to find associations between reason for contact, and 17 other factors, and the probability of alcohol use being checked and overuse tackled.

**Results:**

OHC personnel checked alcohol use twice as often with male patients as with female patients. Employees at risk of needing sick leave were checked for alcohol use more often (55.4, 95% confidence interval 49.2–61.6%) than those on > 30-day sick leave or working with permanent work disability (*p* < 0.01). Alcohol use was checked in 64.1% (59.5–68.7%) of patients while making an individual health promotion plan compared to 36.9% of those without a plan (33.1–40.6%, *p* < 0.0001). Patients with depression were actively checked for alcohol use, especially in cases of major depression (72.7%, 64.0–81.0%). Work processes in which OHC should have been more active in checking and tackling alcohol use included assessing the need for rehabilitation (36.5%, 32.0–41.0%) and health check-ups (HCUs) for mental reasons (43.8%, 38.1–49.4%). HCUs where alcohol overuse was detected led to brief interventions to tackle the overuse in 58.1% (43.4–72.9%) of cases.

**Conclusions:**

The study showed factors that increased OHC personnel’s practices in checking and tackling alcohol use and work processes where the activity should be improved. Discussions about alcohol use took place more often with working-aged men than women, the younger the more. OHC personnel checked actively alcohol use with patients in danger of sick leave, patients treated for depression, while making an individual health promotion plan, and in planned HCUs with a confirmed protocol. More improvement is needed to conduct brief interventions in disability prevention processes, and especially when overuse is detected.

**Supplementary Information:**

The online version contains supplementary material available at 10.1186/s12889-021-12473-2.

## Background

Alcohol use is a risk factor for disability and deaths globally, leading to an estimated 131 million DALYs (disability-adjusted life years) and 3 million deaths annually [1]. *Drinking problems* cover a broad spectrum of practices, from hazardous drinking to harmful alcohol consumption, which can result in physical and psychological impairment, including alcohol dependence. All such drinking patterns, regardless of the degree of severity, are collectively known as alcohol use disorder (AUD). Prevalence estimates range from 4 to 29% of the general population for hazardous drinking and from less than 1 to 10% for harmful drinking [2]. AUD is twice as common among middle-aged men compared to women in the same age group, but only 20% higher among young men compared to young women [3]. The twelve-month prevalence of AUD was approximately 2% in Finland in 2011 [4].

Alcohol overuse is a significant risk factor for early retirement [5]. It is present in more than half of working-aged individuals not participating in education or work [6]. The current research pays close attention to the relationship between AUD and work disability, particularly related to mental disorders [5, 7].

The Alcohol Use Disorders Identification Test (AUDIT-10) was developed to recognize hazardous and harmful drinking [8, 9]. The shorter AUDIT-C questionnaire has also been shown to be valid in the identification of over-consumption [10]. Individuals who exceed the weekly consumption recommendations are at risk of harmful health consequences [9].

Early recognition of excessive alcohol use allows for brief intervention [9]. A meta-analysis of brief interventions in primary care settings found them to be effective [11]. However, brief interventions can be difficult to implement [12]. According to previous studies, people in certain professions are at a higher risk of alcohol-induced morbidity and mortality [13]. In Finland, employees are reached in over 1.4 million health check-ups annually in occupational health care (OHC) [14]. OHC is an ideal setting for alcohol use screening and brief intervention in the case of working-aged people, knowing the hazardous effects of excessive use of alcohol on work ability [15, 16].

Our earlier study showed that activity by OHC personnel to identify excessive use of alcohol was associated with a reduction in disability pensions [17].

## Methods

### Aims

The present study was designed to explore factors, patient characteristics, and work processes in OHC that can explain personnel activity in tackling the overuse of alcohol. Our research question was, “Which patient characteristics and work processes in OHC affect OHC personnel’s activity in their practice of checking patients’ alcohol use and intervening in overuse?”

### Design and setting

Occupational health care in Finland is unique among comparable international contexts: it is integrated with primary health care and is also a health and safety resource provided by the employer within workplaces [18]. Personnel in OHC units in Finland have diverse educational backgrounds. An OHC unit places OHC physicians, OHC nurses, OHC physiotherapists, and OHC psychologists, all of whom have special training for their positions, with assisting personnel in a common work setting. The OHC personnel work in collaboration as a multidisciplinary team, supporting the employer in maintaining a healthy working environment and the employees in staying healthy at work. OHC is strongly regulated by legislation in Finland. According to law, all employees have the right to OHC. We followed the STROBE guideline in performing this study [19].

**Fig. 1:**
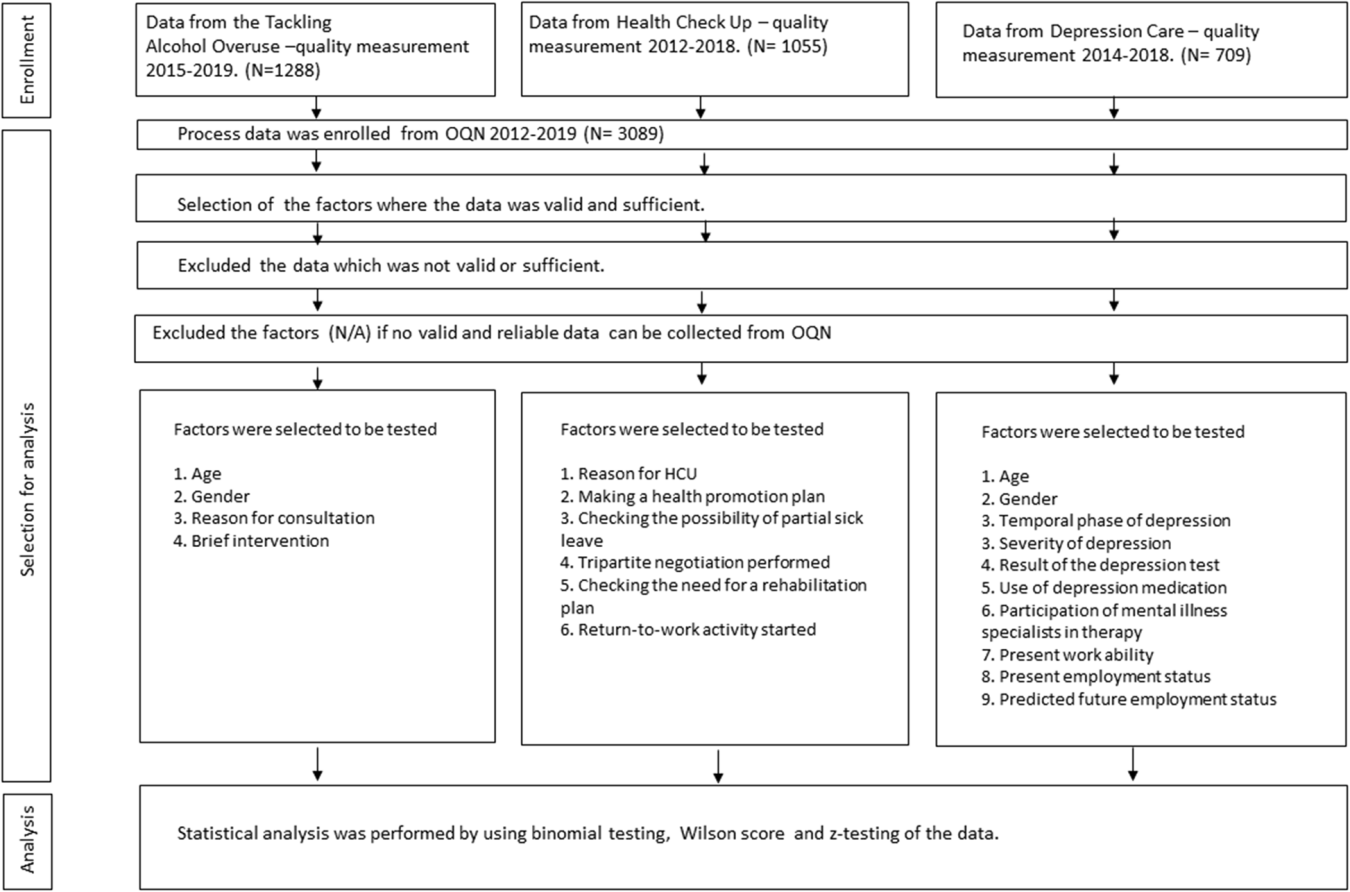
The study overview. The data were from 3089 patient contacts gathered for quality improvement purposes in 15 OHC units during the years 2013–2019 in Finland

This study was designed to use the data collected for quality measurement purposes in the OHC units affiliated with the Finnish Occupational Healthcare Quality Network during 2013–2019 [20]. The data was collected by OHC personnel using structured questionnaires in their patient contacts. The quality measurements were samples of patient flow that were estimated to unveil the needs for improvement in OHC. The collection of data for this study is presented in Fig. 1.

Variables (independent variables) from three different quality measurements that could explain the activity of OHC personnel in checking and tackling alcohol use (the dependent variable) were chosen for the analyses. In some cases quality measurements had identical variables, which allowed us to analyze the association with the variable in different work processes.

#### Data from the tackling alcohol overuse – quality measurement

Data (*N* = 1288) was collected via a questionnaire on the electronic platform annually during 2013–2019 in 15 OHC units. The annual samples were collected over the course of 2 days from all patients consulting the OHC unit. The reason for consultation could be a health check-up (HCU) or medical problem, either mental or physical. An annual sample of 100 patients was considered to strengthen the quality measurement analysis of the OHC unit.

This quality measurement checked OHC personnel’s practices in asking about alcohol use and intervening in cases of overuse. Practices to identify alcohol overuse consisted of briefly asking about alcohol use, counting weekly doses of consumption, or performing AUDIT-10 or AUDIT-C. Four variables were chosen for analysis: age, gender, reason for consultation, and brief intervention.

#### Data from the health check-up – quality measurement

Data (*N* = 1092) was collected via a questionnaire on the electronic platform during 2013–2019 in 15 OHC units. The annual samples were collected over the course of 2 weeks by the OHC personnel performing HCUs. The target population was employees with potential work hazards and those with work ability problems. Data from patients with work ability problems was used in this study. An annual sample of 1–2% of employees registered for OHC was considered to power the quality measurement analyses of the OHC unit.

The quality measurement checked the performance of OHC personnel in the HCUs of employees with reduced work ability, on sick leave or at risk of needing sick leave, and working despite permanent work disability. Six variables were chosen for analysis: the reason for HCU, making a health promotion plan, checking the possibility of partial sick leave, tripartite (i.e., employee, employer, and occupational health care) negotiation performed, checking the need for a rehabilitation plan, and return-to-work activity started.

#### Depression care – quality measurement

Data (*N* = 709) was collected by OHC personnel using a structured questionnaire over the course of 1 month for all consecutive patients with depression during 2014–2019 in 8 OHC units. An annual sample of 50 patients with depression was considered to power the quality measurement analysis of each OHC unit.

The quality measurement checked how OHC personnel followed national guidelines while taking care of employees with depression. Ten variables were chosen for analysis: age, gender, temporal phase of depression (first time, recurrent, or recovery), severity of depression (mild, moderate, or major), result of the depression test, use of depression medication, participation of mental illness specialists in therapy (e.g., consultation with a psychiatrist, psychiatrist therapy, psychotherapy, group therapy, mental illness nurse visits, or psychologist visits), present work ability, present employment status, and predicted future employment status.

Altogether, data from 3089 OHC contacts was analyzed. The association with recognizing alcohol overuse (dependent variable) was tested with age, gender, present work ability, present employment status, predicted future employment status, tripartite negotiation performed, reason for consultation, reason for HCU, making a health promotion plan, assessing the need for rehabilitation, temporal phase of depression, severity of depression, depression test result, use of depression medication, and participation of mental illness specialists in therapy (independent variables). The association with the method of assessing alcohol use was tested for the reason of consultation. The association with the brief intervention to tackle alcohol use was also tested for the reason of consultation.

### Statistical methods

The significance of associations with 95% confidence intervals (CIs) between candidate variables and personnel approach was determined by two-proportion z-test. The Wilson score interval was used in reporting due to its better support of results for proportions near 0 or 1 than the normal approximation interval [21].

If two statistics had non-overlapping confidence intervals, they were significantly different at the 0.05 level. All statistical analyses were performed using the IBM SPSS Statistics 20 software package.

## Results

Patient’s age and gender affected the OHC personnel’s practices in discussing alcohol use. In the Tackling Alcohol Overuse – Quality Measurement data, the activity of thoroughly discussing alcohol use was most frequent with young men (≤35 years of age) (Table 1). The discussion took place twice as often with men as with women in all age groups. In the Depression Care – Quality Measurement data, we did not find significant differences according to age and gender in the activity of checking alcohol use. It was checked with 73.3% (95% CI, 63.3–83.3%) of depressed men and 63.8% (58.6–69.0%) of depressed women during the past year. Comparing patients in age groups with 10-year intervals, activity in checking alcohol use varied from 60.9% (50.7–71.2%) to 79.2% (62.9–95.4%), being most frequent with the youngest and oldest age groups (Supplementary Table 1).

**Table 1:** Employee Demographics and Occupational Health Care Personnel’s Activity in Tackling Alcohol Use

Age and gender	Percentage of total and number	Discussed thoroughly	*p*-value	Started discussion, but alcohol use none or minimal	*p*-value	Did not discuss	*p*-value
Age ≤ 35 men	9.8%	54.0%	Reference	22.2%	Reference	23.8%	Reference
		45.3–62.7%^a^		15.0–29.5%^a^		16.4–31.2%^a^	
	*N* = 126	*N* = 68		*N* = 28		*N* = 30	
Age ≤ 35 women	15.2%	26.0%	**0.004**	35.7%	0.200	38.3%	0.162
		19.9–32.2%^a^		29.0–42.4%^a^		31.5–45.3%1^a^	
	*N* = 196	*N* = 51		*N* = 70		*N* = 75	
Age 36–45 men	5.4%	46.4%	0.484	23.2%	0.942	30.4%	0.604
		34.6–58.1%^a^		13.2–33.1%^a^		19.6–41.3%^a^	
	*N* = 69	*N* = 32		*N* = 16		*N* = 21	
Age 36–45 women	16.5%	18.9%	**< 0.001**	34.9%	0.222	46.2%	**0.031**
		13.6–24.1%^a^		28.5–41.3%^a^		39.5–52.9%^a^	
	*N* = 212	*N* = 40		*N* = 74		*N* = 98	
Age 46–55 men	8.5%	44.5%	0.320	27.3%	0.660	28.2%	0.700
		35.3–53.8%^a^		19.0–35.6%^a^		19.8–36.6%^a^	
	*N* = 110	*N* = 49		*N* = 30		*N* = 31	
Age 46–55 women	20.3%	19.1%	**< 0.001**	33.2%	0.275	47.7%	**0.019**
		14.3–23.8%^a^		27.5–38.9%^a^		41.7–53.8%^a^	
	*N* = 262	*N* = 50		*N* = 87		*N* = 125	
Age ≥ 56 men	7.8%	44.0%	0.309	26.0%	0.748	30.0%	0.593
		34.3–53.7%^a^		17.4–34.6%^a^		21.0–39.0%^a^	
	*N* = 100	*N* = 44		*N* = 26		*N* = 30	
Age ≥ 56 women	16.5%	16.0%	**< 0.001**	43.7%	**0.044**	40.4%	0.107
		11.0–20.9%^a^		37.0–50.3%^a^		33.8–47.0%^a^	
	*N* = 213	*N* = 34		*N* = 93		*N* = 86	

Data taken from the Tackling Alcohol Overuse – Quality Measurement in Finland 2013–2019 (*N* = 1288). The data collection has taken place yearly in all services of occupational health care during a two-week period. The impact of age and gender are analysed having the youngest age group of men as reference

Boldface indicates statistical significance (*p* < 0.05)

^a^Confidence interval = 95%

HCUs due to AUD led to checking alcohol use during the present consultation in 90.5% (81.6–99.4%) of cases, but in HCUs due to mental health problems, musculoskeletal disorders, or without any of the aforementioned reasons, the activity was significantly less frequent, in 38.4% (32.9–43.9%), 44.6% (40.6–48.6%), and 57.1% (51.4–62.8%) of cases, respectively (Table 2). Employees who were at risk of needing sick leave were checked for alcohol use in 55.4% (49.2–61.6%) of cases, and those who had had ≤30 days of sick leave were checked in 54.1% (38.0–70.1%) of cases. Checking alcohol use took place less often in HCUs of employees working despite permanent work disability and employees with longer than 30-day sick leave (Table 3). Making a health promotion plan for the employee during an HCU showed a significant association with checking alcohol use (64.1%, 59.5–68.7%) compared to patients whose health promotion plan was not made (36.9%, 33.1–40.6%). No other support activity of OHC was positively associated with checking alcohol use, and in some support activities, such as checking the possibility of partial sick leave and return-to-work activity started, the association was negative (Table 4).

**Table 2:** Checking Alcohol Use for Employees with Reduced Work Ability

Reason for health check-up	Alcohol use checked in present consultation %	95% CI %	*p*-value	Alcohol use checked in the previous 12 months %	95% CI %	*p*-value
Musculoskeletal disorder (*N* = 585)	44.6 (*N* = 261)	40.6–48.6	**< 0.001**	27.4 (*N* = 160)	23.7–31	**0.035**
Mental health problem (*N* = 297)	43.8 (*N* = 130)	38.1–49.4	**< 0.001**	38.4 (*N* = 114)	32.9–43.9	**< 0.001**
Alcohol overuse (*N* = 42)	90.5 (*N* = 38)	81.6–99.4	**< 0.001**	9.5 (*N* = 4)	0.6–18.4	0.092
None of the diagnosis above (*N* = 289)	57.1 (*N* = 165)	51.4–62.8	Reference	20.8 (*N* = 60)	16.1–25.4	Reference

Data of employees with reduced work ability from the Health Check-up – Quality Measurement 2013–2019 (*N* = 1092). The impact of different diagnosis causing the work disability were compared

Boldface indicates statistical significance (*p* < 0.05)

*CI* Confidence interval

**Table 3:** Checking Alcohol Use for Employees with Work Disability

Reason for health check-up	Alcohol use checked %	95% CI %	*p*-value
Working despite permanent work disability (*N* = 282)	43.3 (*N* = 122)	37.5–49.0	**0.006**
Long sick leave > 90 days (*N* = 120)	32.5 (*N* = 39)	24.1–40.9	**< 0.001**
61–90-day sick leave (*N* = 55)	23.6 (*N* = 13)	12.4–34.9	**< 0.001**
31–60-day sick leave (*N* = 39)	33.3 (*N* = 13)	18.5–48.1	**0.014**
≤30-day sick leave (*N* = 37)	54.1 (*N* = 20)	38.0–70.1	0.877
At risk of needing sick leave (*N* = 249)	55.4 (*N* = 138)	49.2–61.6	Reference

Data from the Health Check-up – Quality Measurement (*N* = 782) 2013–2019. Patients attended the health check-up due to sick leave or they were working despite permanent work disability or they were at risk of needing sick leave

Boldface indicates statistical significance (*p* < 0.05)

*CI* Confidence interval

**Table 4:** Checking Alcohol Use in Various Support Activities of Occupational Health Care

Support activity	Realized activity N	Checked alcohol use %	95% CI %	*p*-value	Not realized activity N	Checked alcohol use %	95% CI %	*p*-value
Making an individual health promotion plan (*N* = 1055)	415	64.1 (*N* = 266)	59.5–68.7	**< 0.0001**	640	36.9 (*N* = 172)	33.1–40.6	Reference
Checking the possibility of partial sick leave (*N* = 251)	210	30.5 (*N* = 64)	24.3–36.7	**0.011**	41	51.2 (*N* = 21)	35.9–66.5	Reference
Tripartite^a^ negotiation performed (*N* = 175)	88	28.4 (*N* = 25)	19.0–37.8	0.705	87	31.0 (*N* = 27)	21.3–40.8	Reference
Assessing the need for rehabilitation (*N* = 496)	438	36.5 (*N* = 160)	32.0–41.0	0.140	58	46.6 (*N* = 27)	33.4–59.7	Reference
Return-to-work activity started (*N* = 175)	133	25.6 (*N* = 34)	18.2–33.0	**0.034**	42	42.9 (*N* = 18)	27.9–57.8	Reference

Data from the Health Check-up – Quality Measurement 2013–2019 (*N* = 1092). Patients attended the health check-up due to sick leave or they were working despite permanent work disability or they were at risk of needing sick leave. The health check-up led to various support activities of occupational health care. The impact of these support activities on checking the alcohol use was analysed

Boldface indicates statistical significance (*p* < 0.05)

^a^Tripartite = employee, employer, and occupational health care provider

The reason for consultation in everyday OHC practice affected the method used in checking alcohol use. AUDIT was used frequently in HCUs of healthy employees, but in contacts where patients had a medical reason for their consultation, either they were only briefly asked about alcohol use or the weekly doses were counted (Table 5).

**Table 5:** Method of Checking Alcohol Use by the Reason for Consultation

Reason for consultation^a^	AUDIT-C or AUDIT-10 performed^b^	Weekly doses counted^b^	Asked briefly about alcohol use	Brief intervention took place during consultation
Health check-up (*N* = 265)	58.9% (*N* = 156)	20.0% (*N* = 53)	18.1% (*N* = 48)	20.4% (*N* = 54)
95% CI	52.8–64.8%	15.2–24.8%	13.5–22.8%	15.5–25.2%
*p*-value	Reference	Reference	Reference	Reference
Medical^c^ (*N* = 106)	8.5% (*N* = 9)	31.1% (*N* = 33)	52.8% (*N* = 56)	34.0% (*N* = 36)
95% CI	3.2–13.8%	22.3–39.9%	43.3–62.3%	24.9–43.0%
*p*-value	**< 0.001**	0.024	**< 0.001**	**0.007**
Mental (*N* = 58)	10.3% (*N* = 6)	34.5% (*N* = 20)	46.6% (*N* = 27)	32.8% (*N* = 19)
95% CI	2.5–18.2%	22.3–46.7%	33.7–59.4%	20.7–44.8%
*p*-value	**< 0.001**	**0.020**	**< 0.001**	**0.046**
Physical (*N* = 34)	2.9% (*N* = 1)	29.4% (*N* = 10)	64.7% (*N* = 22)	41.2% (*N* = 14)
95% CI	0–8.6%	14.1–44.7%	48.6–80.8%	24.6–57.7%
*p*-value	**< 0.001**	0.214	**< 0.001**	**0.010**
Alcohol overuse detected in consultation (*N* = 43)	86.0% (*N* = 37)	4.7% (*N* = 2)	*N* = 0	58.1% (*N* = 25)
95% CI	75.7–96.4%	1.3–15.5%^d^		43.4–72.9%
*p*-value	**0.0014**	**0.019**		**< 0.001**

Data from the Tackling Alcohol Use – Quality Measurement 2013–2019 (*N* = 368). The method of checking alcohol use were compared by the reason of consultation

Boldface indicates statistical significance (*p* < 0.05)

^a^Patients could have multiple reasons for seeking consultation

^b^Two persons were checked for alcohol consumption using both AUDIT and weekly doses

^c^Including physical and mental reasons and patients with partial work ability or need for sick leave

^d^Wilson score interval

*AUDIT* Alcohol Use Disorders Identification Test, CI, confidence interval

A brief intervention to reduce alcohol use was performed among patients who had a medical reason for their consultation in 34.0% (24.9–43.0%) of cases compared to healthy patients in HCU (20.4%, 15.5–25.2%). When alcohol overuse was detected, a brief intervention was performed in 58.1% (43.4–72.9%) of cases (Table 5).

A few variables tested with checking alcohol use among patients with depression had a positive association. The severity of depression affected the checking of alcohol use. Alcohol use was checked in 72.7% (64.0–81.0%) of patients with major depression compared to 56.1% (47.6–64.5%) of patients with minor depression (Supplementary Table 1). The activity of checking alcohol use did not differ between cases of minor depression, moderate depression, recurrent depression, or depression recovery. The patient’s use of depression medication was significantly associated with checking alcohol use among men (77.9%, 69.1–86.7%, *p* = 0.001) but not among women (62.4%, 58.0–66.8%, *p* = 0.187), compared to women without depression medication (56.1%, 47.6–64.5%) (Supplementary Table 1). Checking alcohol use did not differ when a mental illness specialist was participating in therapy compared to no mental illness specialists participating (Supplementary Table 1). Checking alcohol use showed no association with the depression test result, present work ability, present employment status, or predicted future employment status (Supplementary Table 1).

## Discussion

We found several situations in OHC services where the personnel were active in checking and tackling patients’ alcohol use during consultations, but we also found situations where the personnel should have been more active in guaranteeing employees’ health and work ability even by taking up the alcohol use. OHC personnel were more active in discussing alcohol use with men, with younger patients, in planned HCUs with a confirmed protocol, with employees known to overuse alcohol, with employees at risk of needing sick leave, with those who had a health promotion plan under preparation, and with depressed employees, especially when major depression was involved.

Alcohol use was tackled twice as often with men as with women. Since heavy drinking is more common among men than women [7], this is to be expected. The finding that alcohol use discussions took place most often with young men is in accordance with the national health policy to prevent heavy drinking among young people.

We found that in HCUs with a confirmed protocol, checking alcohol use worked well. This highlights the need for fixed protocols in important processes of OHC services [22, 23]. The same positive result of fixed protocols was also seen among patients with depression and excessive use of alcohol. For both patient groups, checking alcohol use is specified in the national guidelines and local procedures. A positive finding was that in two-thirds of the cases, alcohol use was checked when an individual health promotion plan was in preparation, again based on a fixed protocol.

There are several implications of the findings of this study. First, in several situations, discussing alcohol use did not take place as it should in OHC. We were disappointed to note that patients in HCUs due to mental problems were only checked for alcohol use in some 40% of consultations. In everyday practice (i.e., not a planned HCU due to disability) checking alcohol use of patients with mental problems took place even less frequently, although the connection between alcohol use and depression has previously been shown [24, 25]. A study from Finland showed that having an AUD, as opposed to being a light drinker, increased the risk of all-cause sickness absence (hazard ratio, HR = 1.27, 1.04–1.54), but sickness absence due to mental disorders was doubled (HR = 2.16, 1.39–3.35) [7]. The increased risk of work disability caused by excessive alcohol use in cases of mental illness supports checking more actively for alcohol use.

Second, alcohol use should also be checked more actively among patients on a long sick leave. Long sickness absence is a strong predictor of disability pension [26]. Patients who have been on sick leave for 90 days are an important target group for checking alcohol use. In our data, only a quarter of them had been checked for alcohol use, although it is obligatory to get an evaluation of work ability from OHC at the 90-day mark according to the amendment in the Occupational Health Act in 2012 and the demand by the Social Insurance Institution [27]. Other situations where more OHC personnel activity is needed are when preparing for a tripartite negotiation, starting a return-to-work activity, and checking the need for a rehabilitation plan.

Third, the current study showed that further steps are needed to improve checking alcohol use and intervening in excessive use. Alcohol use was tackled with fewer than half of the employees with work disability with medical diagnoses related to alcohol overuse.

If overuse was recognized, a brief intervention to tackle the overuse took place in only 58% of cases. An explanation for this low figure might be that brief interventions are challenging to implement [28]. However, brief interventions in alcohol-related problems have the potential to produce beneficial results [9, 12] and are therefore necessary tools in OHC.

Interestingly, brief interventions in alcohol use took place in some cases despite no overuse being detected. This occurred more often among patients with medical diagnoses compared to those who attended an HCU where no diagnosis was made, suggesting that OHC professionals consider even moderate amounts of alcohol consumption to exacerbate the disease. Another finding was that AUDIT dominated in recognizing the patient’s alcohol use in the HCUs of healthy employees, but in visits for medical reasons, the personnel asked about weekly doses. AUDIT is a fixed procedure in HCUs. On the other hand, asking about weekly doses can give a more concrete basis for treatment discussions about overuse.

It is necessary to recognize alcohol overuse among employees for health risks, disability risks, risks of disability retirement, and increased mortality [1, 2, 5, 13, 29]. The present results demonstrate the importance of implementing fixed protocols in OHC work processes when checking alcohol use and the need to perform brief interventions are crucial. This should take place especially with disability prevention processes. The results show that even in units that have actively worked with work process development, there is a need for improvement.

### Strengths and limitations

The study has several strengths. The study population was sizable and covered employees from all parts of Finland, so the results can be interpreted in most OHC units for local needs. However, the participating units in the quality network are likely to be more active in developing new ways of working, and therefore generalisability of results are limited. Another strength is that the setting provided real-life data with interesting results that can be tested in future research. A third strength is that we were able to test identical variables from different work processes of OHC.

The study also had limitations, including the lack of socioeconomic data for the study population. Since the risk of heavy drinking is greater in some professions, such as manual labor, construction, and service industries [13], this information would have added value to study conclusions. Another limitation was that the data was collected for quality improvement purposes. In some cases, a given answer ruled out subsequent questions, and thus a narrower selection of answers resulted in a smaller sample size for testing particular variables. We found it important, however, that also these results were reported. The insufficient number of observations in some subgroups of employees restricted the statistical analyses, which is a limitation.

The study was an observational study [30], and since this study was conducted in the context of OHC in Finland, the results can to some extent be generalized in Finland, but because of different protocols and guidelines the world over, cautiously in other countries. Ames and Bennett report, on prevention interventions of alcohol problems in the workplace [31]. They conclude that although some studies report significant reductions in alcohol use outcomes, additional research with a stronger and integrated methodological approach is needed. They also suggest that alcohol prevention also might benefit from a guiding framework, which they propose in the article. Our quality improvement efforts where the data originates from included several of the element named in the framework.

Recently a study of clinical guidelines on the management of AUD in Europe was made [32]. Twenty-one European countries have a national guideline for AUD prevention, but still reports of results are scarce.

Finally, in the Tackling Alcohol Overuse – Quality Measurement, the measurement itself may have stimulated the personnel’s activity in asking about alcohol use, creating a possible source of bias.

## Conclusions

Our observational study showed patient characteristics and work processes of OHC that increased the real-life practice of discussing alcohol use and work processes where the practice should be improved. Following patient characteristics, health-related characteristics, and work ability related characteristics increased the practice of tackling the overuse of alcohol; tackling alcohol use took place more often with working-aged men than with women, and more so with younger men. OHC personnel actively initiated the discussion with patients in such care processes as taking care of patients at risk of needing sick leave, treating patients for depression, while making an individual health promotion plan, and in planned HCUs for employees with work disability. More activity to tackle alcohol use is needed with employees on sick leave. This might take place if the discussion is integrated into the fixed protocols of work processes.

## Supplementary Information

**Additional file 1.**

## Abbreviations

AUD: Alcohol use disorder
AUDIT: Alcohol Use Disorder Identification Test
CI: Confidence interval
HCU: Health check-up
HR: Hazard Ratio
OHC: Occupational health care
