# Supplementary material for: Occupational health care personnel tackling alcohol overuse – an observational study of work processes and patient characteristics
Source: BMC Public Health. 2022 Jan 11;22:67. doi: 10.1186/s12889-021-12473-2 (PMC8751364; doi:10.1186/s12889-021-12473-2)
Supplement: Supplementary file 1 — Additional file 1. [file 12889_2021_12473_MOESM1_ESM.docx]

**Supplementary Table 1.** Factors Affecting the Checking of Alcohol Use in Patients with Depression

| Age and gender | Alcohol use checked within 1 year | 95% CI | *p-*value |
| --- | --- | --- | --- |
| ≤29 (N=85) | 73.9% (N=34) | 61%–87% |  |
| 30–39 (N=145) | 60.9% (N=53) | 50.7%–71.2% |  |
| 40–49 (N=214) | 64.6% (N=82) | 56%–73% |  |
| 50–59 (N=219) | 65.3% (N=81) | 57%–74% |  |
| ≥60 (N=46) | 79.2% (N=19) | 62.9%–95.4% |  |
| Men (N=114) | 73.7% (N=56) | 64%–84% |  |
| Women (N=595) | 64.2% (N=213) | 59%–69% |  |
| Type of depression | **Alcohol use checked now** | **95% CI** | ***p*-value** |
| Minor depression (N=152) | 56.1% (N=85) | 47.6%–64.5% | Reference |
| Moderate depression (N=406) | 63.5% (N=258) | 59%–68% | 0.100 |
| Major depression (N=110) | 72.7% (N=80) | 64.0%–81.0% | **0.006** |
| Depression recovery (N=34) | 55.9% (N=19) | 39%–73% | 0.997 |
| Recurrent depression (N=318) | 63.5% (N=202) | 58%–69% | 0.115 |
| Depression test results (MADRS and BDI points) |  |  |  |
| (BDI 30–63, MADRS 31–43) (N=56) | 76.8% (N=43) | 66%–88% | 0.223 |
| (BDI 19–29, MADRS 25–30) (N=99) | 72.7 % (N=72) | 64%–82% | 0.406 |
| (BDI 11–19, MADRS 15–24) (N=66) | 66.7% (N=44) | 55%–78% | Reference |
| (MADRS ≤11 and BDI ≤10) (N=2) | 100.0% (N=2) | 34%–100%^a^ |  |
| Total (N=223) | 72.2% (N=161) | 66%–78% | 0.386 |
| Use of depression medication |  |  |  |
| Men |  |  |  |
| User (N=86) | 77.9% (N=67) | 69.1%–86.7% | **0.001** |
| Non-user (N=28) | 50.0% (N=14) | 31%–69% | 0.563 |
| Total (N=114) | 71.1% (N=81) | 63%–79% | **0.017** |
| Women |  |  |  |
| User (N=463) | 62.4% (N=289) | 58.0%–66.8% | 0.187 |
| Non-user (N=132) | 56.1% (N=74) | 47.6%–64.5% | Reference |
| Total (N=595) | 61.0% (N=363) | 57%–65% | 0.294 |
| Mental illness specialist participating in the treatment |  |  |  |
| Psychiatrist visit (N=204) | 66.7% (N=136) | 60%–73% | 0.150 |
| Psychiatrist consultation (N=42) | 71.4% (N=30) | 32%–59% | 0.163 |
| Therapy visit psychiatrist (N=115) | 64.3% (N=74) | 56%–73% | 0.417 |
| Group therapy (N=8) | 87.5% (N=7) | 52.9%–97.8%^a^ | 0.158 |
| Psychiatric nurse visit (N=156) | 69.2% (N=108) | 62%–76% | 0.066 |
| Psychologist visit (N=334) | 65.9% (N=220) | 60.8%–71.0% | 0.155 |
| Any of the above (N=513) | 63.7% (N=327) | 60%–68% | 0.319 |
| None of the above (N=196) | 59.7% (N=117) | 53%–67% | Reference |
| Present work ability |  |  |  |
| Good work ability (N=230) | 60.9% (N=140) | 55–67% | Reference |
| Minor problems in work ability (N=320) | 60.3% (N=193) | 55%–66% | 0.895 |
| Severe problems in work ability (N=67) | 71.6% (N=48) | 61%–82% | 0.112 |
| Not working due to disability (N=82) | 67.1% (N=55) | 57%–77% | 0.322 |
| Total (N=699) | 62.4% (N=436) | 59%–66% | 0.683 |
| Present employment status |  |  |  |
| At work (N=348) | 61.8% (N=215) | 57%–67% | Reference |
| Partial sick leave (N=36) | 50.0% (N=18) | 34%–66% | 0.177 |
| Sick leave (N=264) | 65.5% (N=173) | 60%–71% | 0.341 |
| Partial provisional pension | NA | NA | NA |
| Provisional pension (N=40) | 62.5% (N=25) | 47%–78% | 0.930 |
| Permanent disability pension (N=10) | 80.0% (N=8) | 49%–94%^a^ | 0.271 |
| Total (N=705) | 62.8% (N=443) | 59%–66% | 0.739 |
| Predicted future employment status |  |  |  |
| At work (N=439) | 60.8% (N=267) | 56%–65% | Reference |
| Partial sick leave (N=49) | 57.1% (N=28) | 43%–71% | 0.620 |
| Sick leave (N=123) | 66.7% (N=82) | 58%–75% | 0.240 |
| Partial provisional pension (N=34) | 55.9% (N=19) | 39%–73% | 0.574 |
| Provisional pension (N=22) | 81.8% (N=18) | 61%–92%^a^ | 0.061 |
| Permanent disability pension (N=12) | 83.3% (N=10) | 55.2%–95.3 %^a^ | 0.142 |
| Total (N=695) | 62.4% (N=434) | 59%–66% | 0.583 |

Data taken from the Depression Care – Quality Measurement in Finland 2013-2019 (N=709). The impact of age, gender, type of depression, depression test results, use of depression medication, participation of mental illness specialist in the treatment, present work ability, present employment status and predicted future employment status on checking the alcohol use was analysed.

*Note:* Boldface indicates statistical significance (*p*<0.05).

^a^ Wilson score interval.

BDI, Beck Depression inventory; CI, confidence interval; MADRS, Montgomery-Åsberg Depression Rating Scale; OHC, occupational health care.
